# Concurrent vitrectomy for persistent pupillary membrane complicated by severe myopia and vitreomacular traction syndrome: A case report

**DOI:** 10.1097/MD.0000000000020895

**Published:** 2020-06-26

**Authors:** Hiroyuki Nishi, Ryohsuke Kohmoto, Masashi Mimura, Masanori Fukumoto, Takaki Sato, Teruyo Kida, Tsunehiko Ikeda

**Affiliations:** Department of Ophthalmology, Osaka Medical College, Takatsuki-City, Osaka, Japan.

**Keywords:** optical coherence tomography, persistent pupillary membrane, vitrectomy, vitreomacular traction syndrome

## Abstract

**Introduction:**

In cases of persistent pupillary membrane (PPM), the eye is usually slightly microphthalmic and emmetropia or hyperopia is often present, yet severe myopia is reportedly rare. Here we presented a case of PPM complicated by vitreomacular traction syndrome and posterior staphyloma due to severe myopia.

**Patient concerns:**

This study involved a 63-year-old female patient who had been diagnosed with bilateral PPM at a local eye clinic and who was subsequently referred to our department for a more detailed examination due to a recent decrease of visual acuity.

**Diagnoses:**

Slit-lamp microscopy examination revealed bilateral PPM. The ocular fundus revealed peripapillary conus and myopic change in both eyes. Optical coherence tomography examination revealed no particular abnormalities in the right eye, yet did show findings indicative of vitreomacular traction syndrome in the left eye.

**Interventions:**

In both eyes, we performed surgical removal of the PPM, phacoemulsification aspiration, and intraocular lens implantation, yet in the patient's left eye, vitrectomy was also performed.

**Outcomes:**

After surgery, the patient's visual acuity improved in both eyes.

**Conclusion:**

The findings in this case show that when required, vitrectomy should be considered based upon the preoperative Optical coherence tomography findings for PPM.

## 1 Introduction

Persistent pupillary membrane (PPM) is sometimes complicated by microcornea, microphthalmia, gonioscopic abnormalities, uveal coloboma, persistent hyperplastic primary vitreous, and congenital cataract. In cases of PPM, the eye is usually slightly microphthalmic and emmetropia or hyperopia is often present, yet severe myopia is reportedly rare.^[1–3]^ Here we report a case of bilateral PPM complicated by severe myopia and vitreomacular traction syndrome (VMTS) in the patient's left eye in which surgical treatment with concurrent vitrectomy resulted in good postoperative outcomes.

## 2 Case report

This study involved a 63-year-old female patient who had been diagnosed with bilateral PPM at a local eye clinic, and who was subsequently referred to our department for a more detailed examination due to a recent decrease of visual acuity (VA).

**Figure 1:**
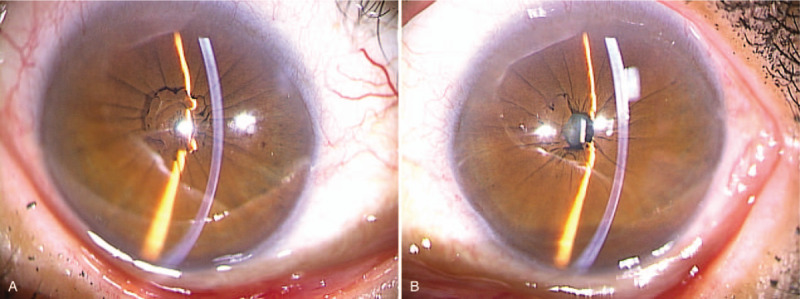
Slit-lamp microscopy photographs of a 63-yr-old female patient with bilateral persistent pupillary membrane (PPM) obtained at the initial examination under non-mydriasis of the pupil. The patient's right eye shows a cauliflower-like PPM and a small pupil area (A). The patient's left eye shows a slightly larger pupil area than that of the right eye, and iris pigment adhered to the anterior surface of the lens can be seen (B).

**Figure 2:**
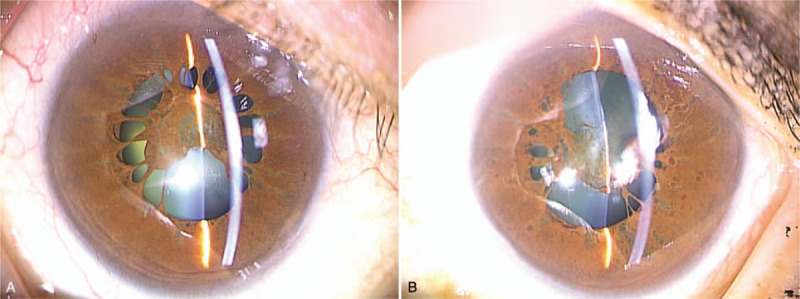
Slit-lamp microscopy photographs of the patient's right and left eyes obtained at the initial examination under mydriasis of the pupil. In both eyes, nuclear cataract can be seen in the lens (A, B).

**Figure 3:**
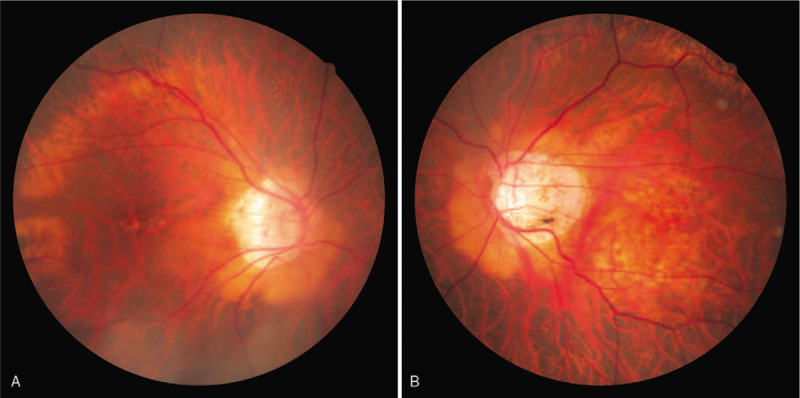
Fundus photographs of the patient's right and left eyes obtained at the initial examination. In both eyes, the ocular fundus, as well as the peripapillary conus and myopic change, can be seen (A, B).

**Figure 4:**
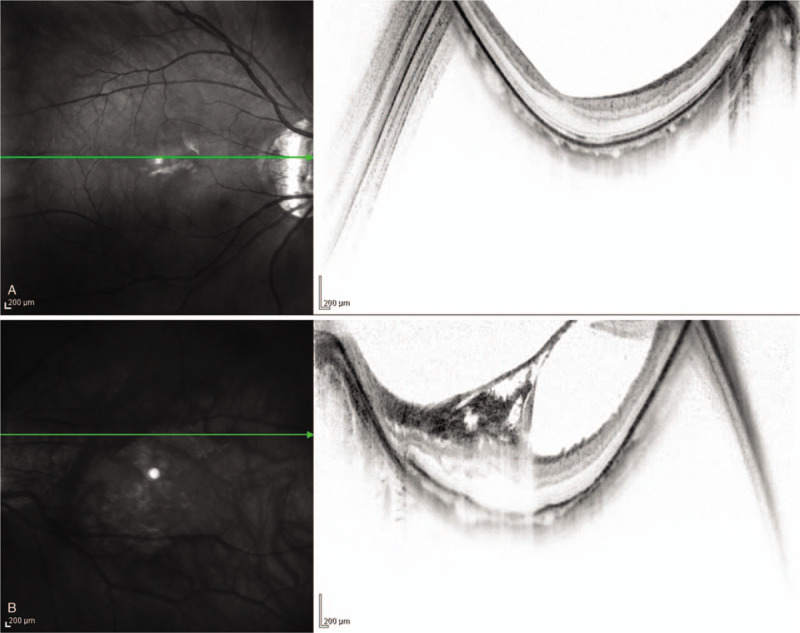
Optical coherence tomography (OCT) images of the patient's right and left eyes obtained prior to undergoing vitreous surgery. In the right-eye image, no particular abnormalities can be seen (A), yet in the left-eye image illustrates findings indicative of vitreomacular traction syndrome (VMTS) (B).

Initial clinical examination revealed that the patient's VA was 0.6 × S – 8.5D = C – 2.5D A × 29° in her right eye and 0.3 × S – 14.0D = C – 3.5D Ax70° in her left eye, and that intraocular pressure in her right and left eye was 17 mm Hg and 14 mm Hg, respectively. Slit-lamp microscopy examination revealed bilateral PPM. Cauliflower-like PPM was observed in her right eye under non-mydriasis of the pupil, and the pupil area was found to be small (Fig. 1A). Although the pupil area in her left eye was slightly larger than that in her right eye, the iris pigment was found to have adhered to the anterior surface of the lens (Fig. 1B). After mydriasis, nuclear cataract was observed in the lens of both eyes (Fig. 2A,B). Moreover, the ocular fundus, as well as peripapillary conus and myopic change, was also observed in both eyes (Fig. 3A,B). The axial length in the patient's right and left eye was 26.34 mm and 27.71 mm, respectively. B-mode ultrasound examination revealed posterior staphyloma in both eyes. Optical coherence tomography (OCT) examination revealed no particular abnormalities in the right eye (Fig. 4A), yet did show findings indicative of VMTS in the left eye (Fig. 4B).

**Figure 5:**
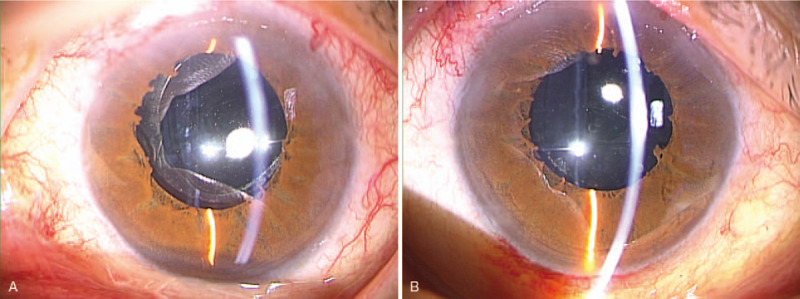
Slit-lamp microscopy photographs of the patient's right and left eyes obtained after surgery. As shown in the images, a normal pupil area was secured in both yes (A, B).

**Figure 6:**
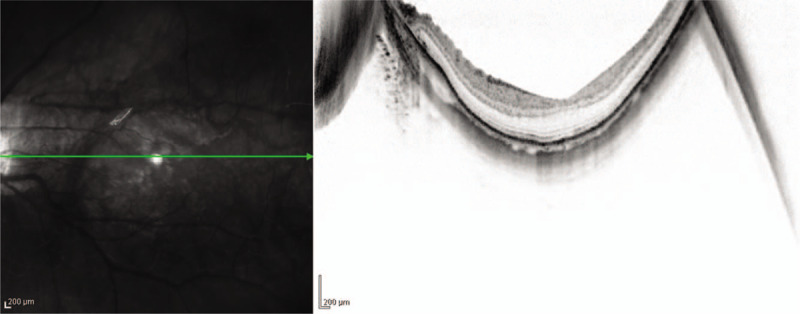
OCT image of the patient's left eye obtained after vitreous surgery. As the image shows, full recovery of the macular shape was obtained. OCT = optical coherence tomography.

Our findings revealed that in the patient's left eye, the deterioration of VA, in addition to the PPM and nuclear cataract, was caused by VMTS. Thus, we first performed surgery on that eye. Briefly, we injected a viscoelastic substance into the anterior chamber, and then removed the PPM with a 25-guage vitreous cutter. At that time, we observed the adhesion of the PPM to the lens at the pigment attachment site on the anterior lens surface. Thus, we subsequently performed phacoemulsification and intraocular lens (IOL) implantation via the standard methods, followed by vitrectomy. The vitreous body was liquefied and degenerated, similar to the condition usually observed in eyes afflicted with severe myopia. The thickened posterior hyaloid membrane was detached and removed from the macular region via the use of vitreous forceps and a diamond-dust scraper, and an artificial posterior vitreous detachment was created toward the periphery. The postoperative course was favorable, and a normal pupil area was secured (Fig. 5B). Moreover, OCT examination revealed recovery of the macular shape (Fig. 6). Corrected VA improved to 0.7. One week later, PPM resection, phacoemulsification, and IOL implantation were performed on the patient's right eye (Fig. 5A), and corrected VA improved to 0.8 post surgery.

This case study was approved by the Ethics Committee of Osaka Medical College, Takatsuki City, Japan, and was performed in accordance with the tenets set forth in the Declaration of Helsinki.

## 3 Discussion

The vascular membrane (tunica vasculosa lentils), which covers the anterior surface of the lens during the fetal period, normally begins to disappear from the 7th month of pregnancy, with complete disappearance usually occurring at around the 9th month. When this absorption is incomplete, mesodermal tissues remain in a filamentous or spider-web-like shape at birth, and this condition is called PPM.^[1–3]^ In such cases, the cord-like substance exits from the collarette of the iris and partially contacts the surface of the lens. The light reflex usually shows no abnormalities, because the lesion site comes out of the collarette of the iris. However, if PPM adheres to the anterior surface of the lens or the pupillary margin adheres, light reflex is impaired at that site. It is usually possible to obtain a good VA from the gap even in the presence of extensive PPM. Thus, the course is often followed up without treatment. However, if VA is affected, laser therapy^[4,5]^ or invasive treatment may be indicated.^[5–7]^ For the laser therapy, the bridge or membranous part of the pupillary membrane is cut via the use of an argon laser or yttrium-aluminum-garnet laser. This procedure can be indicated only if there are no adhesions between the lens and PPM at the site to be irradiated. Invasive treatment includes removal of the PPM with a vitreous cutter. A viscoelastic substance is first injected into the anterior chamber and under the PPM, and the elevated PPM is then resected with a vitreous cutter or vitreous scissors.

As described above, eyes with PPM are often associated with other congenital abnormalities, such as microcornea and microphthalmia. Moreover, the eye is usually slightly microphthalmic, and numerous studies have reported that eye refraction is emmetropia to hyperopia.^[1–3]^ However, and although rare, reports have sporadically appeared of PPM being sometimes observed in cases with severe myopia. In a previou study by Chinta et al., the authors reported a 2-year-old boy with Donnai-Barrow syndrome complicating PPM, revealing that his fundus findings included severe myopia and optic disc hypoplasia.^[8]^ In addition, Gupta et al reported lens extraction and IOL implantation being performed in a 12 year-old boy with PPM complicated by severe myopia and amblyopia.^[9]^ In both of those previous reports, there was no clear description of what caused the severe myopia. However, severe myopia reportedly can be induced by a block of visual stimulation as a result of insufficient retinal image formation due to PPM in childhood.^[10]^ Gupta et al reported that PPM adhered to the anterior capsule of the lens and that iris pigment was attached to the anterior surface of the lens.^[8]^ In our present case, similar findings were observed in the left eye with severe myopia, and the iris pigment on the anterior surface of the lens increased the blockade of visual stimulation. When the patient ultimately reached adult age, axial severe myopia may have progressed accompanied by age-related changes, thereby leading to the development of VMTS. To the best of our knowledge, this is the first report of vitrectomy being performed in a patient with PPM complicated by VMTS.

In conclusion, our findings show that OCT imaging is capable of clearly depicting the state of the fundus even when the pupil is small, and that it also allows the state of the macular region to be easily ascertained after mydriasis even in cases with PPM, like in our present case. Thus, when surgical treatment is performed in patients with PPM, the macular region should be closely examined via OCT imaging prior to surgery, and concurrent vitrectomy should be considered if required.

## Acknowledgments

The authors wish to thank John Bush for editing the manuscript.

## Author contributions

**Conceptualization:** Hiroyuki Nishi, Ryohsuke Kohmoto, Teruyo Kida, Tsunehiko Ikeda.

**Data curation:** Hiroyuki Nishi, Ryohsuke Kohmoto, Masashi Mimura, Masanori Fukumoto, Tsunehiko Ikeda.

**Formal analysis:** Hiroyuki Nishi, Tsunehiko Ikeda.

**Funding acquisition:** Hiroyuki Nishi, Tsunehiko Ikeda.

**Investigation:** Hiroyuki Nishi, Masashi Mimura, Masanori Fukumoto, Takaki Sato, Teruyo Kida, Tsunehiko Ikeda.

**Methodology:** Hiroyuki Nishi, Masashi Mimura, Masanori Fukumoto, Takaki Sato, Teruyo Kida, Tsunehiko Ikeda.

**Project administration:** Hiroyuki Nishi, Tsunehiko Ikeda.

**Resources:** Hiroyuki Nishi, Tsunehiko Ikeda.

**Supervision:** Tsunehiko Ikeda.

**Validation:** Hiroyuki Nishi, Tsunehiko Ikeda.

**Visualization:** Tsunehiko Ikeda.

**Writing – original draft:** Hiroyuki Nishi, Ryohsuke Kohmoto, Tsunehiko Ikeda.

**Writing – review & editing:** Masashi Mimura, Masanori Fukumoto, Takaki Sato, Teruyo Kida, Tsunehiko Ikeda.
